# False Positive Tramadol Urine Testing in Patients Taking Fexofenadine: A Tale of Two Consecutive Cases

**DOI:** 10.1155/2023/4370648

**Published:** 2023-01-05

**Authors:** Suhair Mohammed Yousuf, Ahmad Maaen Alater, Majid Alabdulla, Mugtaba Osman

**Affiliations:** Umm Salal Treatment Centre, Hamad Medical Corporation, Doha, Qatar

## Abstract

Urine drug screen immunoassays have been widely used as point-of-care testing for detection of various drug classes in substance use disorders. However, these immunoassays frequently result in false positive results. We report two patients that used 180 mg daily dose of fexofenadine hydrochloride for treatment of skin allergy and, falsely, tested positive for use of tramadol during urine drug screening. We recommend caution when interpreting positive tramadol urine screening among patients on fexofenadine treatment.

## 1. Introduction

Prevalence of substance use disorder (SUD) is on the rise worldwide, making it a substantial public health concern. Nearly 200,000 deaths were attributed annually to SUD across the globe World Health Organization [1]. The Middle Eastern region serves as a source, consumption market, and transit hub for a range of psychoactive substances ([2]). The State of Qatar lies at the core of the Arab region bordered by other Gulf Cooperation countries. Because of rapid industrialization and economic growth, Qatar has seen a dramatic rise in population. The majority of SUD cases presenting to the emergency department were primarily alcohol-related followed by an array of other psychoactive substances ([3]). Comprehensive treatment packages are now available at addiction and rehabilitation centers across Qatar to help address rising rates of SUD. This system comprises of a stage-based interventional program including inpatient treatment, outpatient follow-up, and aftercare support. Point-of-care urine drug screening tests have been a central component of patient's journey throughout these various stages of treatment ([4]).

Immunoassays help with quick identification of SUD relapse as they provide immediate results within minutes of sample collection. However, they could result in false positive results due to, for instance, structural similarities between related chemical compounds ([5]). Such inaccurate results can be attributed to various factors for example cross reactivity between assay compounds, cut-off concentration levels of drugs and time period between drug ingestion and testing. Ideally, it is recommended that a follow-up chemical test such as gas chromatography–mass spectrometry (GC-MS) or liquid chromatography is performed to get obtain a confirmed analytical urine drug screen result. Unfortunately, due to laboratory limitations, economical setbacks and delay in results GC-MS testing is not widely available in hospitals and rehabilitation centers. Furthermore, it is recommended that faster and more resolving techniques in-lieu of SUD be made available at testing centers, since not all rehabilitation labs have super confirmatory tests like GC-MS or LC-MS-MS ([6]).

False-positive urine drug screening results have profound impact in terms of patient care. Issues with trust-in-care already compromised among SUD patients can become accentuated when false-positive urine results occur. Hence, it is essential that knowledge of such cross-reactions become available and updated, not only for healthcare workers but also for patients and their caregivers.

Fexofenadine, an active metabolite of terfenadine, is a second-generation antihistamine with anti-inflammatory properties with particular antagonism at the H1-receptor [7]. It selectively blocks mast cell histamine release in response to exposure to allergens [8]. Because it binds to these selective receptors, fexofenadine is much less likely to cause drowsiness in comparison to some older antihistamines [9].

To the best of our knowledge, there is no published literature to date on cross-reactivity for fexofenadine with substrates for urine drug screening. Here, we report on two patients with whom such interaction occurred.

## 2. Case Narrative

### 2.1. Case 1

A 40 -year-old male patient was admitted for treatment of alcohol dependence syndrome and bipolar disorder at Umm Salal treatment and rehabilitation center, Hamad Medical Corporation, Doha, Qatar. At the time of admission to inpatient treatment a faStep® multidrug rapid test kit confirmed absence of tramadol in urine samples.

During initial detoxification period, the patient was going through a manic relapse. His initial medication schedule included diazepam, thiamine, and sodium valproate with satisfactory response in terms of withdrawal symptoms from alcohol as well as mood stabilization.

#### 2.1.1. Clinical Findings

Within two weeks of admission, the patient experienced generalized itching and hives, which was diagnosed as idiopathic urticaria. This necessitated a course of fexofenadine hydrochloride 180 mg daily in addition to topical hydrocortisone and emollients.

#### 2.1.2. Diagnostic Assessment

A routine urine drug screening was carried out within three days post fexofenadine initiation. The tested positive for tramadol on faStep® multidrug rapid test kit. Patient categorically denied use of any illicit substances. A subsequent test was done after three more days, and the results remained consistently positive for tramadol. Due to laboratory limitations, a confirmatory GC/MS testing was not available. Simultaneously, a thorough investigation of the patient's room, belongings, and a safely environmental check was done, and no contrabands were found. A detailed discussion was carried out with the patient following the second set of results and he confirmed nonuse of tramadol. Patient was understandably frustrated, and his mood deteriorated.

#### 2.1.3. Therapeutic Intervention

Fexofenadine was discontinued as it was the only new medication that was added to his medication schedule and the patient was started on another second-generation antihistamine—levocetirizine dihydrochloride—5 mg daily.

#### 2.1.4. Follow-Up and Outcomes

A urine drug screening, under strict supervision, was performed one-week post fexofenadine discontinuation and it was negative from tramadol on all the subsequent samples.

#### 2.1.5. Patient Perspective

Patient expressed delight and content with confirmation of nonuse of tramadol.

### 2.2. Case 2

A 23-year-old male patient was admitted for treatment of polysubstance abuse at Umm Salal treatment and rehabilitation center, Hamad Medical Corporation, Doha, Qatar.

Patient underwent an initial comprehensive detoxification phase of two weeks followed by the rest of days in rehabilitation phase. At the time of admission to inpatient treatment, a faStep® multidrug rapid test kit confirmed absence of tramadol in urine samples.

#### 2.2.1. Clinical Findings

During his stay in rehabilitation phase, patient experienced itching and hives on his upper extremities, diagnosed as dermatitis. This necessitated a two week of course fexofenadine hydrochloride 180 mg daily in addition to topical mometasone and emollients.

#### 2.2.2. Diagnostic Assessment

A routine urine drug screening was carried out within five days post fexofenadine initiation. The patient tested positive for tramadol on faStep® multidrug rapid test kit. Patient categorically denied use of any illicit substances. A subsequent test was done after nine more days, and the results remained consistently positive for tramadol. Due to laboratory limitations, a confirmatory GC/MS testing was not available. Simultaneously, a thorough investigation of the patient's room, belongings, and a safely environmental check was done, and no contrabands were found. A detailed discussion was carried out with the patient following the second set of results and he confirmed nonuse of tramadol.

#### 2.2.3. Follow-Up and Outcomes

A urine drug screening, under strict supervision, was performed at the end of fexofenadine course and it was negative for tramadol on all the subsequent samples.

#### 2.2.4. Patient Perspective

Patient expressed delight and content with confirmation of nonuse of tramadol.

## 3. Discussion

Reports of false negative urine screening results in the context of SUD have been circulating in the literature throughout the last two decades ([10]). Reports of such diagnostic inaccuracy kept trickling until recently with strong detrimental effect on therapeutic alliance and doctor-patient trust ([11]). Agreement between self-reported use and urine-confirmed drug use results was lowest for opioids ([12]), hence compromising an indispensable reliance on urine testing for detection of such substances.

Tramadol is a quasi-narcotic synthetic analgesic that acts primarily through low agonism of mu opioid receptors ([13]), and is used in the treatment of moderate to severe pain with consistent effectiveness ([14]). Tramadol prescription rose substantially in primary care and pain management settings, occasionally for prolonged durations and not in keeping with therapeutic guidance ([15]). Therefore, patients were put at risk of iatrogenic dependence. Tramadol use amongst SUD population has become a major health concern, particularly in the Arabian Gulf region ([16]).

On the other hand, false positive results for tramadol were reported in the literature for venlafaxine; an antidepressant medication of the serotonin-norepinephrine reuptake inhibitor class. That was linked to similar immunoassay transition times for both tramadol and venlafaxine when medical scientists use the “liquid chromatography linked to atmospheric pressure ionisation tandem mass spectrometry” method for detection of tramadol in urine ([17]). Evidence also exists for cross reactivity between phencyclidine and tramadol in terms of urinary samples investigated using immunoassay methods ([18]). According to manufacturer's information, a general cut-off concentration of 300 ng/mL was set for detection of tramadol. However, no specific information was available with regards to sensitivity, specificity, or predictive values.

Tramadol distribution includes all body compartments and is 20% protein-bound with principal elimination through the urinary system and 6-hour half-life ([19]). Notably, tramadol is detectable in urine samples for up to four days post-ingestion.

No scientific case reports or case series, to the best of authors' knowledge, were found in the literature regarding cross reactivity between fexofenadine and tramadol in terms of urine drug testing. A United Kingdom-based drug testing kit's manufacturer has placed an alert of potential fexofenadine-induced false positive screening for tramadol; however, no thorough description of patients was provided ([20]). Our patients displayed clear and temporal associations between intake of fexofenadine 180 mg per day and positive tramadol urine test. Notably, the tests were negative for tramadol once fexofenadine was stopped.

A limitation of our report is the difficulty to generalize results based on only two cases. There is a minimum likelihood that our patients may still have taken tramadol despite their verbal assurances and lack of circumstantial evidence. Moreover, our results may not apply to urine samples tested by techniques other than faStep® multidrug rapid test kit. Another significant limitation is the lack of confirmatory testing procedure in our health facility at the time. We contacted our laboratory department to secure a tramadol blood level. However, it was not feasible logistically at the time.

There are still limitations that no confirmatory test has been performed on the two patients described in this manuscript. Moreover, no test has been done with fexofenadine in a nonabuser. If such a test would have been performed it would be quite clear that the test can give a false result when fexofenadine is used by the patient, even in the absence of a confirmatory test. Without this information the generalization of the results is not evident.

A further investigation of interest to validate our conclusion would be to test a person, known not to be an SUD patient, on fexofenadine for presence of tramadol in urine. Hence, one important focus for further research is to investigate patients taking fexofenadine at variable doses in terms of presence of urine evidence for tramadol. Furthermore, commonly prescribed antihistamines should also be investigated to evaluate their effects on tramadol urine drug screening. We recommend caution when interpreting positive tramadol urine screening among patients on fexofenadine treatment.

## 4. Conclusion

Fexofenadine is widely prescribed for allergy symptoms, urticaria and dermatitis. It can be procured as an over-the-counter medication. False-positive results for tramadol in patients on fexofenadine is extremely rare and challenging to identify as literature review on the subject to the best of our knowledge, has not been previously reported. Hence, it is important that clinicians are aware of this interference.
